# Atf4 regulates angiogenic differences between alveolar bone and long bone macrophages by regulating M1 polarization, based on single-cell RNA sequencing, RNA-seq and ATAC-seq analysis

**DOI:** 10.1186/s12967-023-04046-1

**Published:** 2023-03-14

**Authors:** Lanxin Gu, Zhongyuan Wang, Hong Gu, Hua Wang, Luwei Liu, Wei-Bing Zhang

**Affiliations:** 1 Department of Orthodontics, The Affiliated Stomatological Hospital of Nanjing Medical University, Nanjing, 210029 China; 2 Jiangsu Province Key Laboratory of Oral Diseases, Nanjing, 210029 China; 3 Jiangsu Province Engineering Research Center of Stomatological Translational Medicine, Nanjing, 210029 China; 4 Department of Urology, The First Affiliated Hospital of Nanjing Medical University, Nanjing, 210029 China; 5 Nanjing Stomatological Hospital, Medical School of Nanjing University, Nanjing, 210029 China; 6 Department of Stomatology, Dushu Lake Hospital Affiliated to Soochow University, Suzhou, China; 7 Department of Stomatology, Medical Center of Soochow University, Suzhou, China; 8 Department of Stomatology, Suzhou Dushu Lake Hospital, Suzhou, China

**Keywords:** Single-cell sequencing, ATAC-seq, Angiogenesis, Macrophage, Transcription factors

## Abstract

In the repair of maxillofacial bone defects, autogenous craniofacial bone can often provide superior clinical results over long bone grafts. Most current studies have focused on the osteogenic differences between alveolar bone marrow (ABM) and long bone marrow (LBM), however, studies about the angiogenic differences between the two are currently lacking. We downloaded single-cell RNA sequencing (scRNA-seq) of mouse ABM and LBM respectively from the public database, and the data were processed by using Seurat package. CellphoneDB2 results showed that macrophages had the strongest interaction with mesenchymal stem cells (MSCs) and endothelial cells (ECs). ELISA results confirmed that ABM macrophages secreted a higher level of vascular endothelial growth factor A (Vegfa) compared to LBM macrophages, which further promoted angiogenesis of ECs and MSCs. Using SCENIC package, six key transcription factors (TFs) were identified to regulate the difference between ABM and LBM macrophages, and activating transcription factor 4 (Atf4) was confirmed to be more expressed in ABM macrophages by polymerase chain reaction (PCR) and western blot (WB), with predicted target genes including Vegfa. Besides, the result of scRNA-seq implied ABM macrophages more in M1 status than LBM macrophages, which was confirmed by the following experiments. From the results of another assay for transposase accessible chromatin sequencing (ATAC-seq) and RNA-seq about M1 macrophages, Atf4 was also confirmed to regulate the M1 polarization. So, we suspected that Atf4 regulated the different expression of Vegfa between ABM and LBM macrophages by activating M1 polarization. After knocking down Atf4, the expression of M1 polarization markers and Vegfa were downregulated and vasculogenic differences were eliminated, which were subsequently reversed by the addition of LPS/IFN-γ. Our study might provide a new idea to improve the success rate of autologous bone grafting and treatment of oral diseases.

**Supplementary Information:**

The online version contains supplementary material available at 10.1186/s12967-023-04046-1.

## Introduction

Autogenous bone grafts are commonly used to reconstruct maxillofacial bone defects due to congenital malformations, tumors, injuries, and infections. Craniofacial bone grafts could offer superior clinical results than long bone grafts in repairing maxillofacial bone defects [1]. Several studies indicated that significant osteogenic difference existed between alveolar bones and long bones. The proliferation rate of alveolar bone mesenchymal stem cells (BMSC) was greater than those of BMSC of long bone [2, 3]. Alveolar bone cells expressed more alkaline phosphatase and calcium deposition than long bone cells [3]. Considering that blood vessels could supply the nutrient requirements for bone remodeling and precursor cells for osteoblasts and osteoclasts, angiogenesis also plays a key role in bone restoration and remodeling [1]. However, the differences in angiogenesis between alveolar and long bone are currently lacking in research.

Traditional transcriptome analysis is conducted on mixed cell population without sufficient resolution to identify specific cell clusters [4]. Single-cell RNA sequencing (scRNA-seq) allows for the analysis of organizational heterogeneity at the individual cell levels and the exploration of the roles of different cell clusters on physiological functions and pathogenesis [5]. Three articles have revealed the bone marrow microenvironment of mouse long bones at single-cell resolution [6–8]. The atlas of the immune microenvironment of mouse mandibular alveolar bones has been constructed by scRNA-seq and this study has also confirmed the osteogenic difference between mouse alveolar bones and long bones at single-cell resolution [5].

Bone marrow is a complex microenvironment of multiple cell types including various hematopoietic stem cells (HSCs), bone marrow/lymphoid progenitor cells and immune cells. These cells interact with each other to regulate the homeostasis of the bone system [9]. Macrophages, as key mediators of the immune system, play important roles in the destruction and rebuilding of bone tissues [10], and their regulatory role of angiogenesis in bone remodeling has been demonstrated by promoting the differentiation of mature ECs [11]. The polarization status of macrophages could affect angiogenesis and osteogenesis. Early activation of proinflammatory (M1) macrophages is essential for cell infiltration and initial angiogenesis, whereas subsequent activation of anti-inflammatory (M2) macrophages favors new bone formation and enhances bone-implant integration in the later stages of bone restoration [12, 13]. One study revealed alveolar bone marrow (ABM) macrophages more in M1 polarization status than long bone marrow (LBM) macrophages, which might lead to the difference of osteogenic effects of these macrophages on BMSC [5]. However, it has not been investigated whether the different polarization states of macrophages at these two sites result in different abilities to promote angiogenesis.

In this study, with the help of scRNA-seq, we found that ABM macrophages promoted angiogenesis than LBM macrophages by secreting more vascular endothelial growth factor A (Vegfa). By interacting single-Cell rEgulatory Network Inference and Clustering (SCENIC) and assay for transposase-accessible chromatin using sequencing (ATAC-seq), the difference of angiogenesis might be caused by differences in the proportion of M1 macrophages at these two sites regulated by activating transcription factor 4 (Atf4).

## Methods

### The scRNA-seq data of ABM and LBM processing

We obtained the fastq.gz data of ABM in the European Nucleotide Archive (ENA) database (PRJNA697839) and the data of LBM in the Gene Expression Omnibus (GEO) database (GSE109774), and used cellranger count (v 1.1.0) to get the raw single-cell gene expression matrix. Applying the Seurat package (v 4.0.1), we further combined the matrix with the metadata to form the object. Genes expressed in more than three cells and cells expressed with more than 300 genes were included. Cells with more than 25% mitochondrial genes were excluded. “LogNormalize”, the default global-scaling normalization, was performed with a scale factor setting of 10,000 to get the normalized genes. Then the function (FindVariableGenes) was then used to pick out variable genes. Principal component analysis (PCA) was then applied, based on the results of which UMAP and tSNE dimensionality reduction was conducted. After that, clusters were obtained and annotated respectively according to the original literature [5, 14]. In addition, to make the macrophage cluster in ABM and LBM comparable, we used the function (FindIntegrationAnchors and IntegrateData) to remove the batch effect.

### Ligand–receptor interactions

Since CellPhoneDB2 only allowed for the import of human genes, we transferred the mouse gene symbols to the responding human gene symbols using the BioMart package [5]. Cell–cell communication was later conducted to find the potential ligand-receptor relationships between cell clusters through CellPhoneDB2 [15]. Only the ligand-receptor relationships (P-values < 0.05) were taken into account, and the ligand-receptor pairs were sorted according to their mean values of expression.

### SCENIC analysis of macrophages of ABM and LBM

The SCENIC analysis was applied to explore key transcription factors regulating difference between ABM and LBM macrophages. The SCENIC analysis was conducted through R package SCENIC (v 1.1.0.1) with default parameters following the standard procedure [16]. Firstly, we filtered the recombined expression matrix of ABM and LBM macrophages based on RcisTarget's database (mm9-tss-centered-10 kb) and then calculated the correlation and normalize the matrix. Then, GEne Network Inference with Ensemble of trees (GENIE3) was applied to infer co-expressed modules between TFs and potential target genes. After that, the inferred group of all TFs included was considered as GRNs using CisTarget [17], while all genes with corresponding TFs patterns in their regulatory space were identified as valid TF targets. Lastly, the regulators in each cell were scored by AUCell [16] and the regulator activity (AUC) scores were mapped to the heat map to visualize.

### ATAC-seq and RNA-seq of M1 macrophages

The processed expression matrix of RNA-seq of mouse M1 bone marrow-derived macrophages (BMDM) were from GSE183565 (GSM5560756, GSM5560757, GSM5560758, GSM5560765, GSM5560766, GSM5560767). Deseq2 (FDR < 0.05 and |fold change|> 1.5) was used to calculate differentially expressed genes (DEGs) between the M1 and untreated BMDM.The raw ATAC-seq data were downloaded from the ENA database (PRJNA761334) (GSM5560738, GSM5560739, GSM5560740, GSM5560747, GSM5560748, GSM5560749). As for ATAC-seq, Trim Galore! was used to filter the adaptor sequences. The trimmed reads were mapped to mm10 build genome by Bowtie2 and were transferred to bam form. Then, polymerase chain reaction (PCR) duplicates, reads mapping to mitochondrial DNA, as well as low quality reads were obviated to get high quality sequences by SAMtools. MACS2 was applied to call peaks and irreproducible discovery rate (IDR) was calculated to evaluate the repetition between samples. All sequencing tracks were viewed using the Integrated Genomic Viewer (IGV 2.3.61). Later, Diffbind (v 3.0.13) by employing DESeq2 was conducted with FDR < 0.05 and |fold change|> 1.5 to reveal the differentially expressed peaks between the M1 and the control BMDM, which were annotated by ChIPseeker (v 1.26.0) later. We also used tagHeatmap and plotAvgProf function to visualize the distribution of the peaks near promoters. To combine ATAC-seq and RNA-seq, we annotated genes from peaks with increased openness in the ATAC-seq and intersected them with up-regulated genes found in the RNA-seq to select genes. Later, the selected genes were reversed to corresponding peaks, and then we used HOMER function findMotifsGenome.pl. on the selected peaks to find Motifs to which specific TFs could bind.

### Isolation and culture of BMSCs and ECs

The femur and tibia of 12-week-old male C57BL/6 mice (from Nanjing Medical University Experimental Animal Center, Jiangsu, China) were separated. Washed out by complete culture medium: 10% FBS (ScienCell), 1% penicillin/streptomycin (Hyclone) in LG-DMEM (Gibco), bone marrow was transferred to a 100 mm diameter petri dish and was cultured at 37 °C, 5% CO_2_ for 2 days. The suspended cells were collected, centrifuged at 1000 rpm for 5 min, and further cultured in EGM-2MV medium (Lonza) on the petri dish pre-coated with 10 μg /mL human fibronectin (Gibco) to get ECs. And the adherent cells (BMSCs) were cultured with complete culture medium [18]. The medium was changed every two days and digestion was performed when the confluence was 80% for passaging.

### Isolation of BMDM and collection of conditioned medium (CM)

ABM and LBM macrophages were separated from 12-week-old male C57BL/6 mice. The detailed protocol could be found in the raw literature [5]. Separated ABM and LBM macrophages were cultured at the same density in complete culture medium with 50 ng/mL M-CSF (R&D Systems) added [19]. The culture media were collected every three days and were centrifuged at 1000 rpm for 10 min to get the supernatant as CM. Vegfa levels of CM were quantified by Vegfa ELISA Kit (Cusabio, China) following the manufacture protocol. CM were mixed with complete culture medium or EGM-2MV in a ratio of 1:1 respectively for BMSCs and ECs. BMSCs at Passage 3 were seeded in 6-wells plates with CM at 1*10^5^/well, with 2 replicate wells in each group for three weeks for subsequent experiments.

### siRNA transfection

The sequences of siRNA against Atf4 (OBiO Technology, China) were 5ʹ-GGAUGUUGGAGAAAAUGGATT-3ʹ (sense) and 5ʹ-UCCAUUUUCUCCAACAUCCTT-3ʹ (antisense) and the sequences of negative control were 5ʹ-UUCUCCGAACGUGUCACGUTT-3ʹ (sense) and 5ʹ-ACGUGACACGUUCGGAGAATT-3ʹ (antisense). Macrophages were transfected with siRNA by Lipofectamine 2000 (Invitrogen, USA) according to the protocol of the manufacture.

### Cell immunofluorescence staining

BMSCs were seed in 24-wells plates at 2*10^4^/well, with 2 replicate wells in each group. After 24 h culture, cells were washed twice with PBS for 5 min each time. Samples were fixed with 4% paraformaldehyde at 4 ℃ for 30 min, permeabilized with 0.5% Triton X-100 for 10 min (not for macrophages), blocked with goat serum (BOSTER) for 30 min. BMSCs were incubated with rabbit anti-Cd34 (Affinity, 1:100). After overnight incubation, samples were incubated with anti-rabbit 488-labeled antibody (Proteintech, 1:50) for 60 min, counterstained with DAPI (VECTASHIELD, 1:50) for 2 min. The pictures were observed with the immunofluorescence microscope (Olympus, Japan).

### Tube formation assay

ECs and BMSCs were placed on 96-wells plates pre-coated by matrigel (BD Biosciences, 356231) at 2*10^4^ cells/well with three replicates per group, and were incubated with their respective CM at 37 ℃, 5% CO_2_ for 12 h. Tube formation results were recorded by an inverted microscope and the number of meshes were quantified using the Image J software.

### PCR analysis

RNA from macrophages was isolated by Trizol reagent (Invitrogen, USA) and then was reverse transcribed into cDNA using Primescript RT Master Mix kit (Takara, Japan). The relative transcription levels were calculated by assessed by the ABI QuantStudio 7 Flex (Thermo Fisher, USA). The primers were listed in Additional file 1: Table S1.

### Western blot (WB)

Proteins were obtained by RIPA lysis buffer (Beyotime, China) and the concentrations were assessed by BCA assay (Beyotime, China). After being separated by 10% SDS-PAGE, proteins were transformed onto polyvinylidene fluoride membranes (Millipore, Billerica, MA). Membranes were blocked with 5% skim milk for two hours and then incubated with the following primary antibodies overnight at 4 °C: anti-Atf4 (Abcam, 1:1000), anti-Vegfa (Proteintech, 1:1000), anti-iNOS (Abcam, 1:1000), anti-Arg1(Abcam, 1:1000), anti-β-actin (Proteintech, 1:1000). After three washes, the membranes were incubated with secondary antibodies for one hour at room temperature, and the blots were captured by Tanon 5200 luminous imaging system. The results were quantified using the Image J software.

### Statistical analysis

Data were shown as mean ± SEM. Two-tailed Student’s t-test was applied to compare the difference between two groups and one-way or two-way analysis of variance (ANOVA) with Tukey’s post hoc test was used for multiple comparisons. All experiments were all repeated three times. Corrected P-values less than 0.05 were considered statistically significant.

## Results

**Fig. 1:**
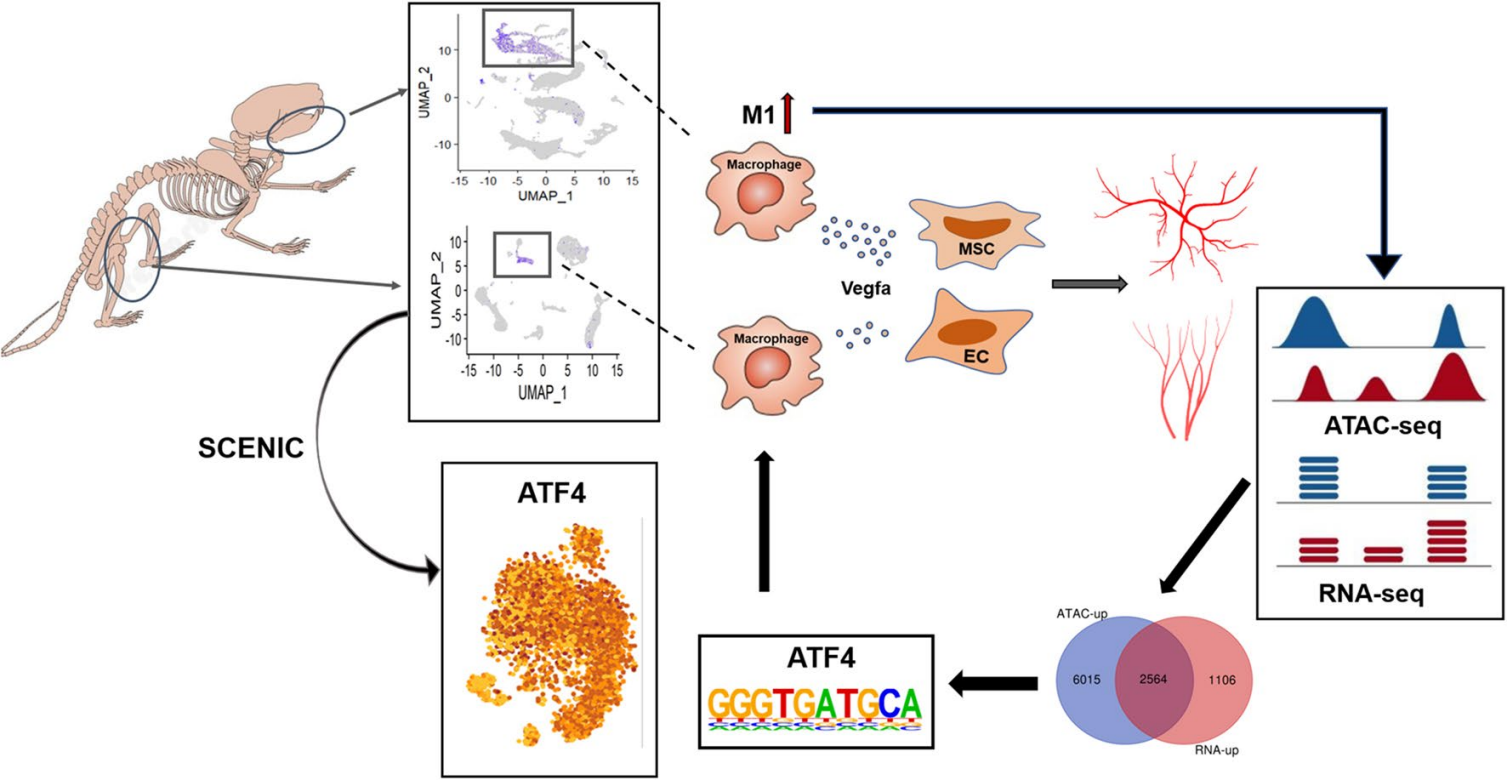
Flow chart of the study

The flow chart of our study has been demonstrated in Fig. 1.

### Processing and characteristics of scRNA-seq of ABM

**Fig. 2:**
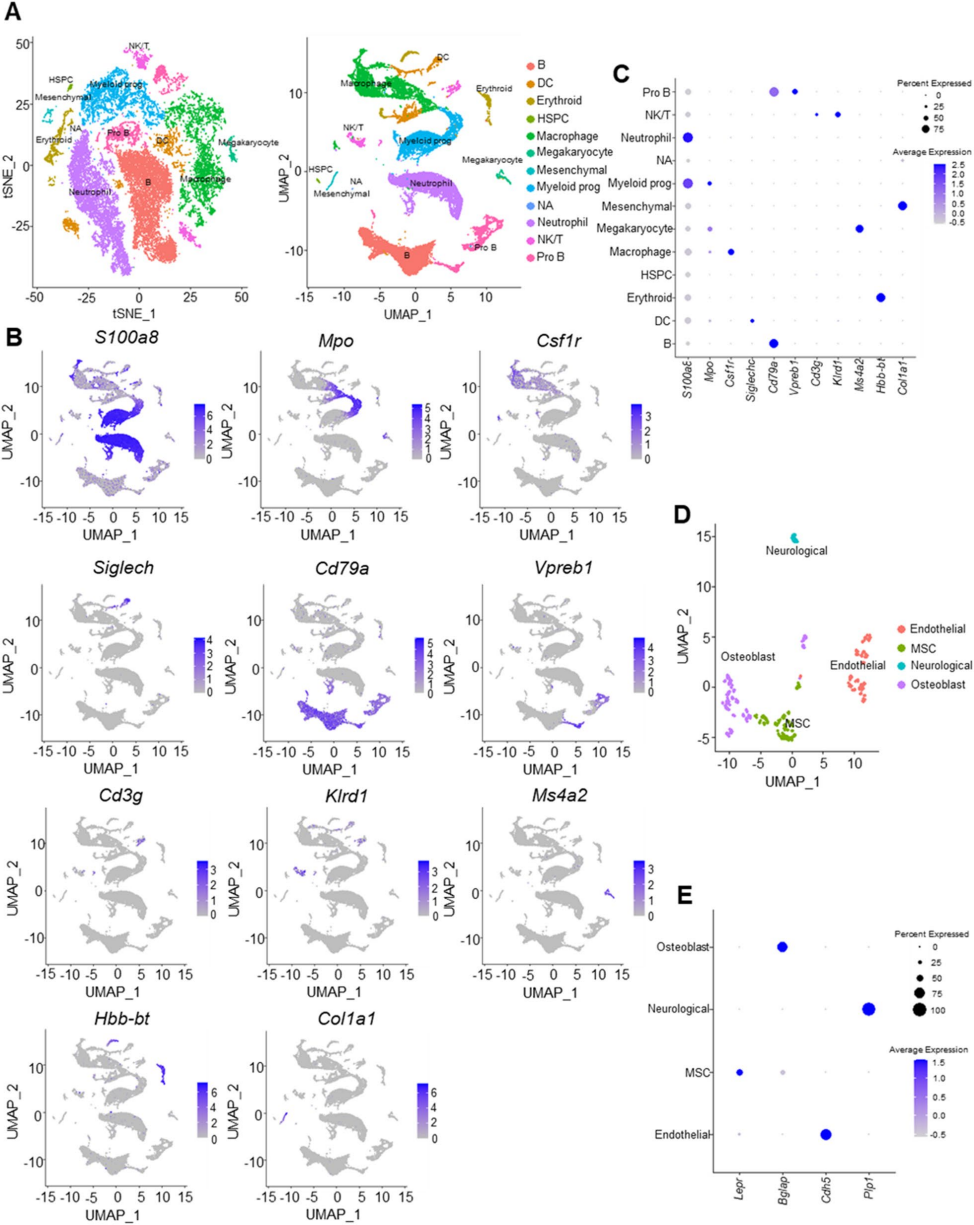
Characterization of ABM single-cell atlas. **A** Cells identified by scRNA-seq were visualized with Tsne (left) and UMAP (right). Different cell populations were defined and distinguished by color. Each point represented an independent cell. **B** The expression levels of cell markers were projected onto UMAP atlas. **C** Specific expression of cell markers in different cell types. **D** Cells identified from mesenchymal cells were visualized with UMAP; **E** Specific expression of cell markers in the four stromal cell subtypes. *ABM* alveolar bone marrow, *MSC* mesenchymal stem cell

We combined the raw counts of the four ABM samples in the beginning and as shown in Additional file 1: Fig. S1A, the reproducibility between the four samples was good and there was almost no batch effect. So, we directly used the combined data for subsequent analysis. And the distribution of data after quality control was shown in Additional file 1: Fig. S1B. Using “FindVariableFeatures” function, 2000 variable features were singled out from 18,445 features and top 10 were highlighted on the Additional file 1: Fig. S1C. As shown in Fig. 2A, applying tSNE and UMAP methods, we divided these cells into 11 clusters and annotated these cell clusters based on the classical cell surface markers (Table 1), and the expression of markers in different cell type clusters was shown in Fig. 2B, C. Using “FindAllMarkers” function, 5734 highly variable genes were singled out and top 5 genes from each cluster were demonstrated in Additional file 1: Fig. S1D. Then the mesenchymal cell cluster was isolated and was divided into four subclusters (Lepr for MSCs, Bglap for osteoblasts, Cdh5 for ECs, and Plp1 for neurological cells), as shown in Fig. 2D, E.

**Table 1:** Annotated cell subpopulations and cell surface markers

Cell type	Marker
Neutrophil	S100a8
Myeloid progenitor	Mpo
Macrophage	Csf1r
Dendritic cell	Siglech
B cell	Cd79a
Pro-B cell	Vpreb1
T cell	Cd3g
Natural killer cell	Klrd1
Megakaryocyte	Ms4a2
Erythrocyte	Hbb-bt
Mesenchymal cell	Col1a1

### Ligand–receptor interactions analysis

**Fig. 3:**
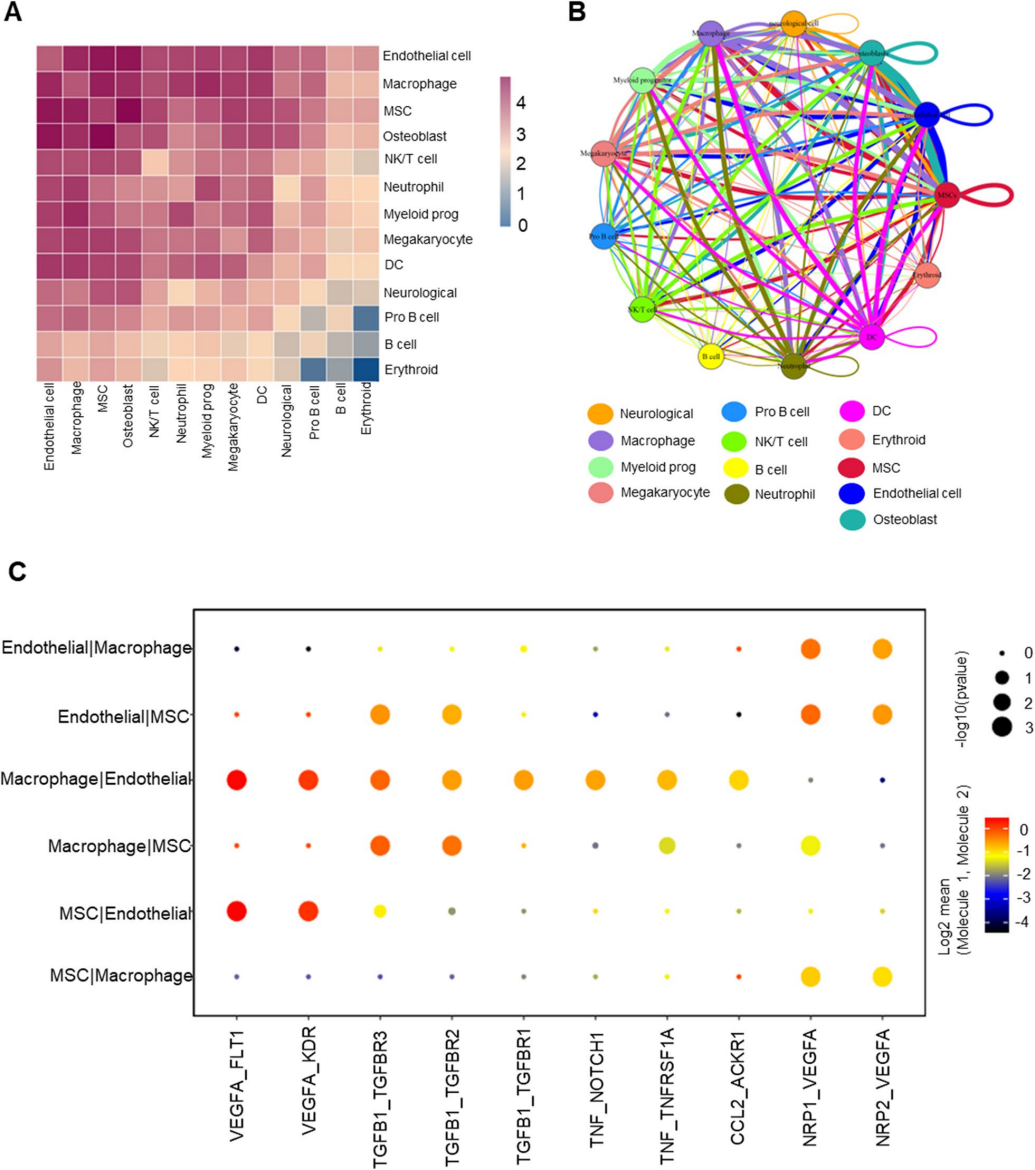
Cell–cell interactions among different cell types in ABM. **A** Heatmap of the cell–cell interactions. **B** Network diagram of the cell–cell interactions of different cell types in ABM. **C** Visualization of the selected ligand–receptor interactions between macrophages, MSCs, ECs. *ABM* alveolar bone marrow, *MSC* mesenchymal stem cell, *EC* endothelial cell

From the results of pheatmap, macrophage had a wide range of interactions with other cells, with the strongest interactions with MSCs and ECs (Fig. 3A). The network of interactions between 13 cell subclusters was shown in the circus plot (Fig. 3B). Further, we explored the important cytokines engaged in the interactions between macrophages, MSCs and ECs and plotted them according to their mean values of expression (Fig. 3C). Vegfa-Nrp1/Nrp2 and Tgfb1-Tgfbr2/Tgfbr3 were predicted in the macrophage-MSC interactions, and Vegfa-Kdr/Flt1, Tgfb1-Tgfbr1/Tgfbr2/Tgfbr3, Tnf-Tnfrsf1a/notch1, Ccl2/Ackr1 were predicted in the macrophage-EC interactions. Briefly, macrophages secreted Vegfa, Ccl2, Tnf, Tgfb1 to interact with MSCs and ECs.

### Processing of LBM scRNA-seq and the comparation between ABM and LBM

**Fig. 4:**
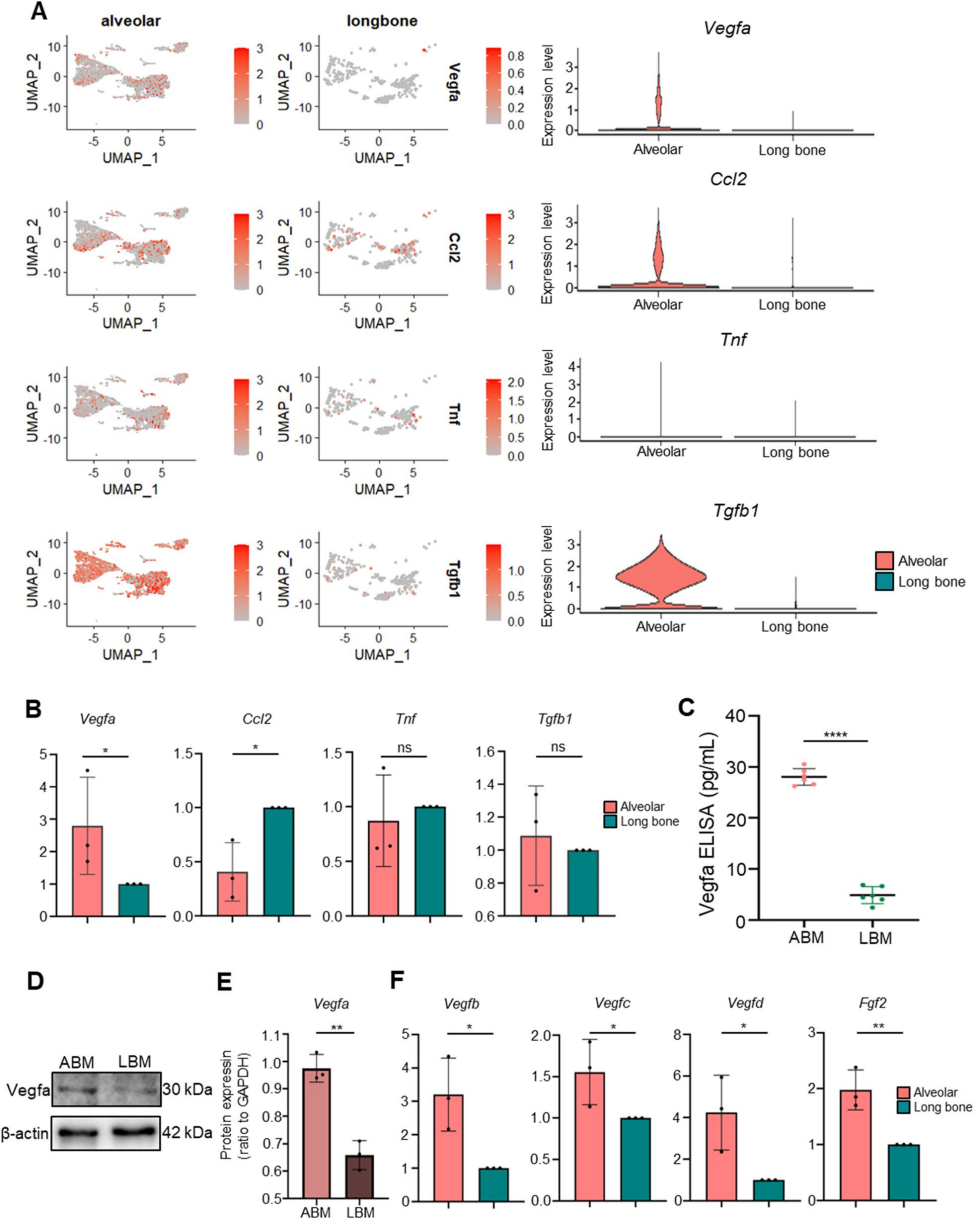
Vegfa with high expression in ABM macrophages. **A** Expression of the selected four ligands secreted by ABM macrophages and LBM macrophages. **B** Expression of the four ligands in ABM macrophages and LBM macrophages was analyzed by PCR assay. **C** ELISA for Vegfa in ABM and LBM conditioned medium. **D** Comparison of Vegfa expression in ABM macrophages and LBM macrophages by WB. **E** Quantification of the expression of Vegfa in ABM macrophages and LBM macrophages. **F** Expression of Vegfb, Vegfc, Vegfd, Fgf2 in ABM macrophages and LBM macrophages was analyzed by PCR assay. *Corrected P-value < 0.05. **Corrected P-value < 0.01. *ABM* alveolar bone marrow, *LBM* long bone marrow, *Vegfa* vascular endothelial growth factor A, *Vegfb* vascular endothelial growth factor B, *Vegfc* vascular endothelial growth factor C, *Vegfd* vascular endothelial growth factor D, *Fgf2* fibroblast growth factor 2, *PCR* polymerase chain reaction

To expose the unique features of macrophages of ABM, a new scRNA-seq data of LBM was used. Quality control and dimensionality reduction were conducted and five clusters were obtained (Additional file 1: Fig. S2A). Using the macrophage markers “Ahnak”, “Mpeg1”, “Cd68”, “Csf1r” [14], macrophage cluster (cluster 3) was separated and the spots were plotted in Additional file 1: Fig. S2B. Later, we used function (FindIntegrationAnchors and IntegrateData) to remove the batch effects and combined ABM macrophage cluster with LBM macrophage cluster (Additional file 1: Fig. S2C, D). The distributions of the above key cytokines (Vegfa, Ccl2, Tnf, Tgfb1) in ABM and LBM macrophage were shown in Fig. 4A, demonstrating that the four cytokines expression in ABM macrophages was higher than that in LBM macrophages. From the results of PCR (Fig. 4B), only the level of Vegfa were statistically higher in ABM macrophages than in LBM macrophages. ELISA result further confirmed that Vegfa content of ABM macrophage CM was higher than that of LBM macrophage CM (Fig. 4C). WB result also showed the protein content of Vegfa was higher in ABM macrophages than in LBM macrophages (Fig. 4D, E). Considering Vegfa was strongly associated with angiogenesis and macrophages were closely interacted with MSCs and ECs, we suspected that ABM macrophages played a more pro-angiogenesis role in MSCs and ECs than LBM macrophages. At the same time, we also explored the expression of other angiogenetic factors [20] in ABM and LBM macrophages and the PCR results showed that the expression of Vegfb, Vegfc, Vegfd, and Fgf2 was all significantly higher in ABM macrophages than in LBM macrophages (Fig. 4F), which further indicated ABM macrophages might be more capable of promoting angiogenesis than LBM macrophages.

### Transcriptional regulators analysis and polarization status difference between LBM and ABM macrophages

**Fig. 5:**
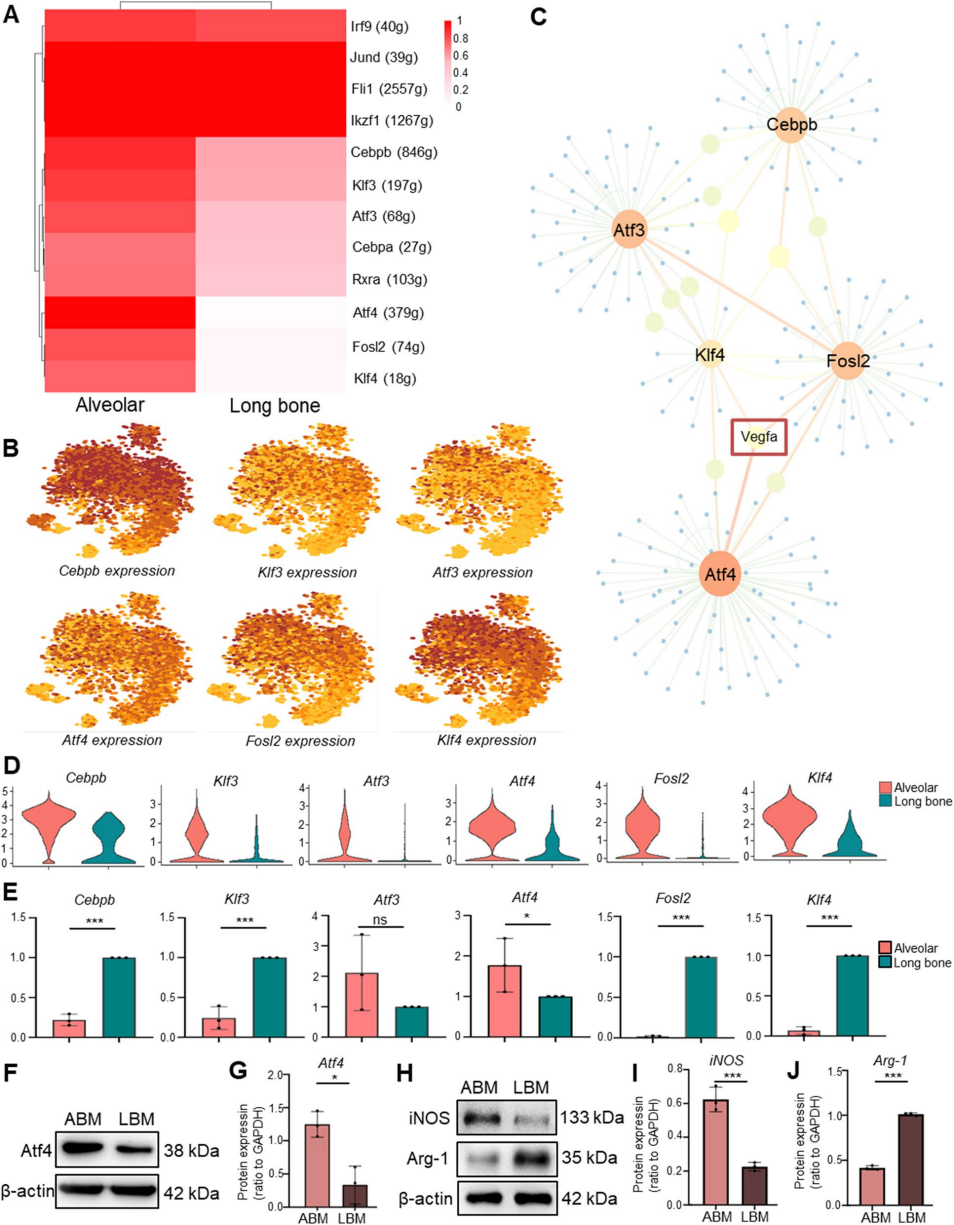
Identification of TF–target gene interactions in ABM and LBM macrophages using SCENIC and polarization status difference between the two. **A** The heatmap showed the enrichment of TFs of ABM and LBM macrophages. **B** In the tSNE dimension, the darker the AUC of the selected six TFs in each cell cluster, the higher the AUC value. **C** TF–target gene interaction network. **D** Expression of the six TFs in ABM macrophages and LBM macrophages. **E** Expression of the six TFs in ABM macrophages and LBM macrophages was analyzed by PCR assay. **F** Comparison of Atf4 expression in ABM macrophages and LBM macrophages by WB. **G** Quantification of the expression of Atf4 in ABM macrophages and LBM macrophages. **H** Comparison of iNOS, Arg-1, expression in ABM macrophages and LBM macrophages by WB. **I** Quantification of the expression of iNOS in ABM macrophages and LBM macrophages. **J** Quantification of the expression of Arg-1 in ABM macrophages and LBM macrophages. Data were expressed as mean ± SEM from 3 independent experiments. *Corrected P-value < 0.05. ***Corrected P-value < 0.001. *TF* transcription factor, *ABM* alveolar bone marrow, *LBM* long bone marrow, *AUC* area under the curve, *Atf4* activating transcription factor 4, *iNOS* inducible nitric oxide synthase, *Arg-1* arginase-1, *PCR* polymerase chain reaction, *WB* western blot

We used SCENIC analysis to explore potential TFs leading to the difference between ABM macrophages and LBM macrophages. The heat map results showed that Cebpb, Klf3, Atf3, Atf4, Fosl2, and Klf4 were more active in ABM macrophages than in LBM macrophages (Fig. 5A). Figure 5B demonstrated the cell activity distributions of the six regulators in the tSNE dimension. The network showing the six regulators and their respective target genes was plotted and Vegfa was found to be co-regulated by Klf4, Fosl2, and Atf4 (Fig. 5C). The distribution of the six regulators in ABM and LBM macrophages were shown in Fig. 5D and these regulators were all more expressed in ABM macrophages than in LBM macrophages. However, the further PCR results confirmed that only the level of Atf4 was significantly higher in ABM macrophages than in LBM macrophages (Fig. 5E), which was also verified by WB (Fig. 5F, G). Since the macrophage polarization status is highly correlated with angiogenesis [21], we suspected the difference between macrophages in the two positions was due to different polarization status. As shown in Additional file 1: Fig. S2E, the M1 polarization markers (Cd86, Tnf, Nos2) were more significantly enriched in ABM macrophages than in LBM macrophages and the M2 polarization marker (Arg1) was the opposite, implying that ABM macrophages were more in the M1 status. From the results of WB, the protein content of iNOS (M1 polarization marker) was higher in ABM macrophages than in LBM macrophages and the Arg1 protein level was the opposite (Fig. 5H, I, J).

### ATAC-seq and RNA-seq analysis of M1 macrophages

**Fig. 6:**
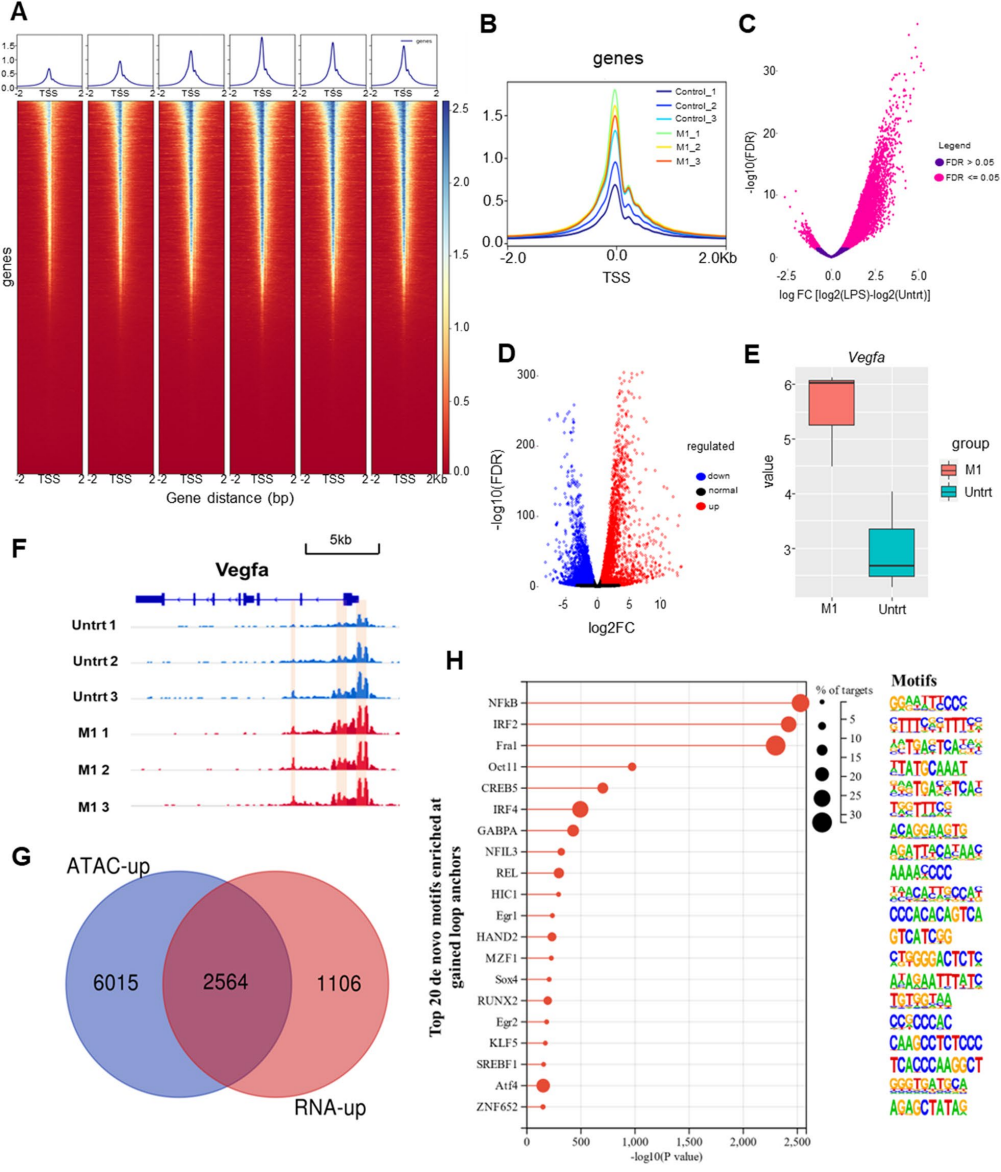
ATAC-seq and RNA-seq analysis of mouse M1 BMDM. **A** Separated heatmaps and average intensity profiles of promoter accessibility over all annotated TSSs in the mouse genome. **B** Combined average intensity profiles of promoter accessibility over all annotated TSSs. **C** The volcano-plot of differential accessible ATAC-seq peaks comparing LPS-stimulated BMDM (M1) and untreated BMDM. **D** The volcano-plot of differentially expressed genes comparing LPS-stimulated BMDM and untreated BMDM in RNA-seq results. **E** Expression of Vegfa in LPS-stimulated BMDM and untreated BMDM in RNA-seq results. **F** Localization of Vegfa gene in the chromatin to show its different accessibility of promoters between LPS-stimulated BMDM and untreated BMDM. **G** Venn diagram of the genes annotated from more accessible peaks in ATAC-seq and the up-regulated genes in RNA-seq comparing LPS-stimulated BMDM and untreated BMDM **H** Top 20 de novo motifs enriched in the more accessible peaks with up-regulated expression comparing LPS-stimulated BMDM and untreated BMDM. *BMDM* bone marrow-derived macrophages, *TSS* transcription start sites, *Vegfa* vascular endothelial growth factor A

To further explore the TFs regulating the M1 polarization of macrophages, we downloaded the ATAC-seq and RNA-seq data of the 50 ng/mL LPS stimulated (M1 polarization) mouse BMDM. PCA plots of ATAC-seq and RNA-seq revealed a strong correlation between replicates of the same group and the comparable correlations between the untreated group and the LPS-stimulated group (Additional file 1: Fig. S3A). Figure 6A demonstrated that most accessible areas were identified in 2 kb of transcription start sites (TSS), suggesting that accessible regions of chromatin participated in transcriptional regulation. And the accessibility of TSS in BMDM was increased significantly under LPS stimulation (Fig. 6A, B). The peaks in the promoter regions (3 kb upstream and downstream of TSS) accounted for 35.73%, 35.4%, and 35.2% of the whole area respectively for the three samples of untreated BMDM, and 45.02%, 38.5%, and 35.73% respectively for the three samples of M1 BMDM (Additional file 1: Fig. S3B–D), also showing that M1 BMDM had higher transcriptional activation than the untreated BMDM. Processing data by Diffbind, we found 19,021 peaks in promoter region representing the chromatin more accessible in M1 BMDM than in untreated BMDM and 793 peaks less accessible in M1 BMDM (Fig. 6C). As for the results of RNA-seq, there were 7404 genes differentially expressed in M1 BMDM compared with untreated BMDM, 3670 (49.57%) of which were up-regulated and 3734 (50.43%) of which were down-regulated (Fig. 6D). Vegfa was also found up-regulated in the RNA-seq (Fig. 6E), and its chromatin openness was also increased (Fig. 6F), meaning that M1 polarization stimulated chromatin opening of Vegfa, leading to its increased expression. Then, the 8579 genes annotated from the 19,021 peaks with increased openness were intersected with the 3670 up-regulated genes obtained by RNA-seq to obtain 2564 genes whose expression was increased due to the enhances chromatin openness under the LPS stimulation (Fig. 6G). De novo motif analysis was conducted on the 8050 peaks from which 2564 genes were annotated and 39 TFs were found. The top 20 were demonstrated in Fig. 6H. Considering the results together with SCENIC results, we hypothesized that Atf4 as a potential TF could regulate the angiogenic difference between ABM and LBM through regulating M1 polarization.

### Difference of angiogenic effects of ABM and LBM macrophages

**Fig. 7:**
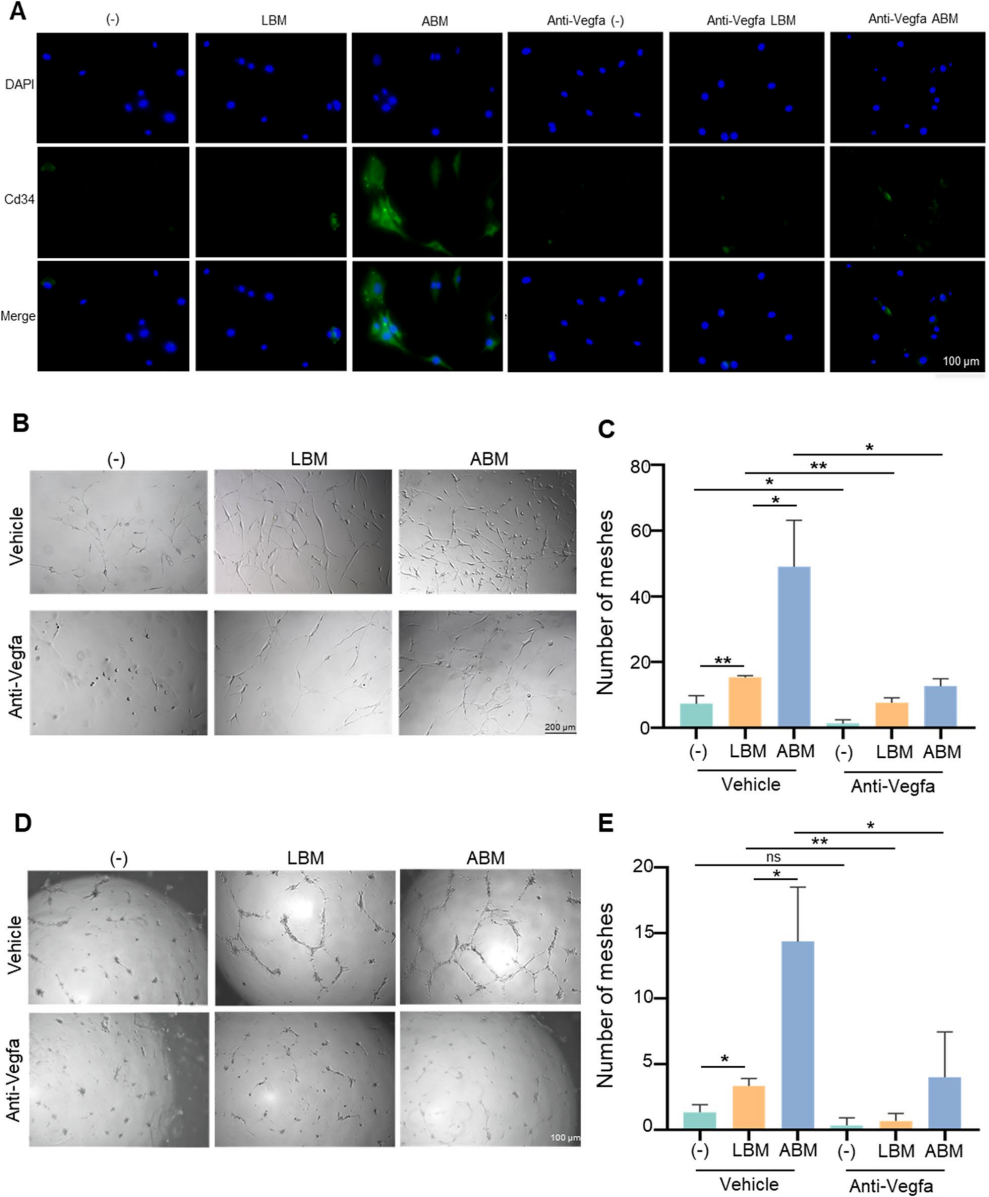
Difference of angiogenic effects of ABM and LBM macrophages. **A** DAPI (blue), Cd34 (green) immunofluorescence staining (scale bar, 100 μm). **B** Tube formation effect of macrophage CM on BMSCs (scale bar, 200 μm). **C** The plot of statistical analysis for numbers of meshes of BMSCs. **D** Tube formation effect of macrophage CM on ECs (scale bar, 100 μm). **E** The plot of statistical analysis for numbers of meshes of ECs. Data were expressed as mean ± SEM from 3 independent experiments. *Corrected P-value < 0.05. **Corrected P-value < 0.01. *ABM* alveolar bone marrow, *LBM* long bone marrow, *CM* conditioned medium, *BMSC* bone mesenchymal stem cells, *EC* endothelial cell

Both ABM and LBM CM promoted MSCs transforming into ECs with the representative marker of Cd34 and the effects of ABM CM tended to be more significant (Fig. 7A). In the tube formation analysis, both CM could promote tube formation and the promotive effect of ABM was more significant (Fig. 7B), paralleled by increased number of meshes (Fig. 7C). As for the ECs, ABM macrophages also lead to more tube formation than LBM macrophages (Fig. 7D, E). After Vegfa neutralization, ABM and LBM CM showed similar ability to promote the transformation of MSCs to ECs (Fig. 7A) and tube formation (Fig. 7B–E).

### Atf4 was a potential TF to regulate the angiogenic difference between ABM and LBM through M1 polarization

**Fig. 8:**
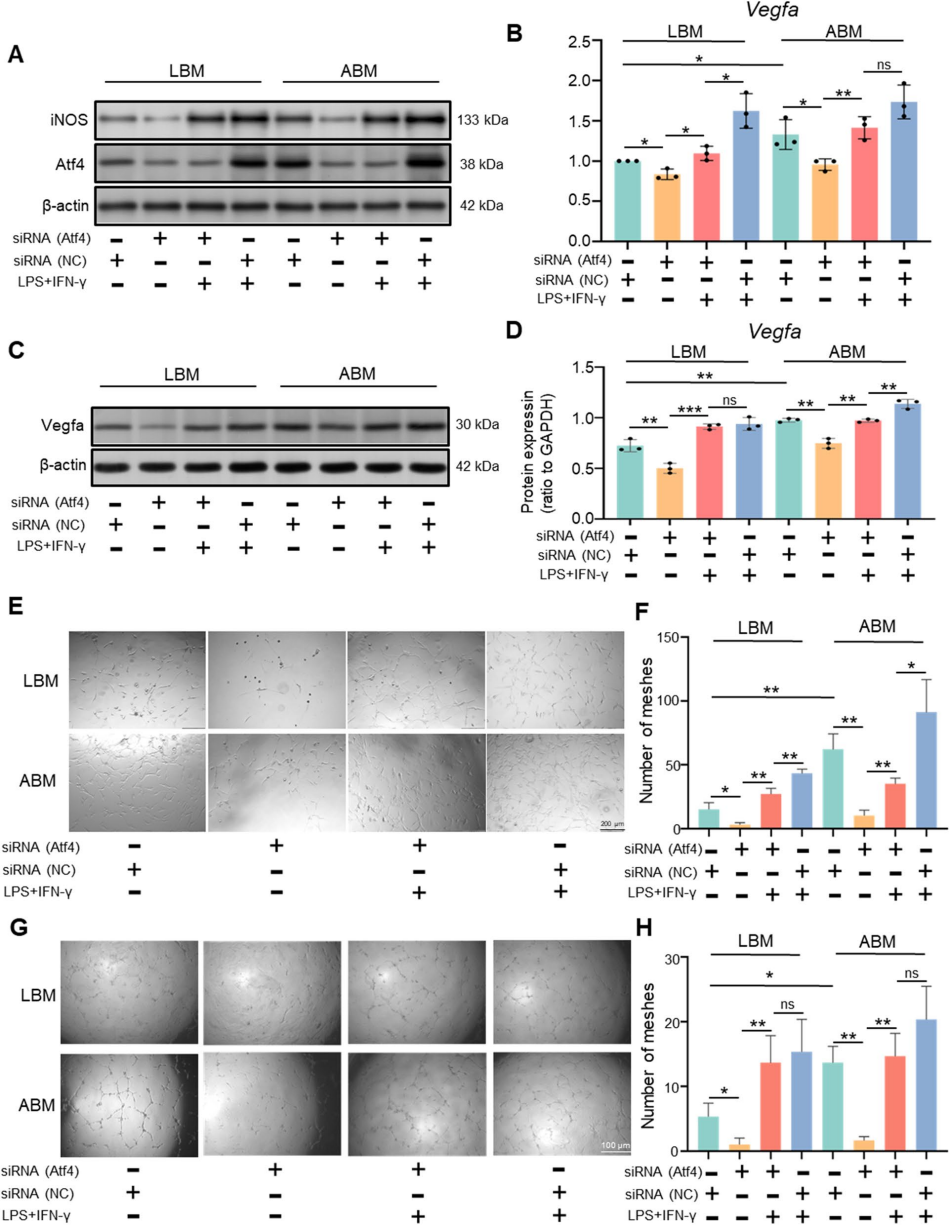
Atf4 regulating the angiogenic difference between ABM and LBM macrophages through M1 polarization. **A** WB analysis of iNOS and Atf4 in different groups. **B** PCR analysis of Vegfa in different groups. **C** WB analysis of Vegfa in different groups. **D** Quantification of the expression of Vegfa in ABM macrophages and LBM macrophages. **E** Tube formation effect of different CM on BMSCs (scale bar, 200 μm). **F** The plot of statistical analysis for numbers of meshes reflecting different effects of CM on BMSCs. **G** Tube formation effect of different CM on ECs (scale bar, 100 μm). **H** The plot of statistical analysis for numbers of meshes reflecting different effects of CM on ECs. *Corrected P-value < 0.05, **Corrected P-value < 0.01, ***Corrected P-value < 0.001. *iNOS* inducible nitric oxide synthase, *Atf4* activating transcription factor 4, *Vegfa* vascular endothelial growth factor A, *ABM* alveolar bone marrow, *LBM* long bone marrow, *WB* western blot, *PCR* polymerase chain reaction, *CM* conditioned medium, *BMSC* bone mesenchymal stem cells, *EC* endothelial cell

When adding siRNA depleting Atf4 to ABM and LBM macrophages, the expression of iNOS was significantly reduced (Fig. 8A) and the polarization difference between ABM and LBM was reduced. PCR result (Fig. 8B) and WB result (Fig. 8C, D) also demonstrated that Vegfa expression was decreased by inhibiting Atf4. When adding 50 ng/mL LPS and 300ug/mL IFN-γ into the cultural medium of macrophages which could promote M1 polarization, the expression of repressed Atf4, iNOS and Vegfa was reversed (Fig. 8A–D). The tube formation of MSCs (Fig. 8E, F) and ECs (Fig. 8G, H) was also inhibited by siRNA (Atf4), which was also reversed by additional LPS and IFN-γ. Besides, additional add of M1 polarization factor could eliminate the angiogenic difference between ABM and LBM macrophages (Fig. 8E–H).

## Discussion

Autogenous bone grafts are commonly used to repair maxillofacial bone defects and craniofacial bone grafts could offer superior clinical results than long bone grafts in repairing maxillofacial bone defects [1]. Most current studies have focused on the osteogenic differences between alveolar bones and long bones. However, the angiogenic differences between the two are currently lacking in research. Considering angiogenesis plays a key role in bone remodeling [1], we used scRNA-seq, ATAC-seq, and RNA-seq to explore the angiogenic difference between ABM and LBM and the potential TFs to regulate such difference.

From the results of ligand–receptor interaction analysis, macrophages showed the strongest interaction with MSCs and ECs, and Vegfa, secreted by macrophages, was significantly more expressed in ABM macrophages than in LBM macrophages. Vegfa is a potent inducer of angiogenesis because it binds highly specific to the tyrosine kinase receptor of ECs thereby promoting proliferation, migration and neointima formation [22], and it could also induce differentiation of MSCs to ECs to promote angiogenesis. We found that ABM macrophages CM enhanced ECs angiogenesis directly and by promoting the differentiation of BMSCs into ECs thus promoting angiogenesis more than LBM macrophage CM, which could be inhibited by adding anti-Vegfa antibody, implying that the angiogenic difference was related to the different Vegfa content in the two conditioned media. There are several possible reasons for the difference in Vegfa content between these two sites. Firstly, the mechanical load on the alveolar bones is twice the one on the long bones [23], which has been proven to activate the expression of Vegfa and promote angiogenesis [24]. Secondly, mandibular alveolar bones tend to ossify in an intramembranous manner, whereas long bones tend to ossify in a cartilaginous way [25]. Differences in angiogenic capacity are indeed associated with different origins and osteogenic patterns [26], and Vegfa is particularly highly expressed in the mesenchyme that gives rise to the skull and mandible, while it is less expressed in cartilage structures [27]. Thirdly, due to long-term exposure to the oral microbial environment, inflammatory responses are active in alveolar bones [28], and inflammation could significantly activate Vegfa expression and angiogenesis [29], which might lead to the different Vegfa content in ABM macrophage and LBM macrophage. Fourthly, both mechanical force [30] and inflammatory response have been demonstrated to promote macrophage polarization toward M1 status, which could also account for our finding that ABM macrophages are more in M1 status than LBM macrophages. And M1 polarization has been confirmed to promote the expression of Vegfa [31].

As we all know, besides Vegfa, the VEGF protein family also included Vegfb, Vegfc, Vegfd, and so on, which all have angiogenesis potential [32]. Vegfb is particularly able to promote vascularization in cardiac and skeletal muscle in both adult and embryonic tissues [33]. Vegfc and Vegfd play major roles in promoting lymphatic vessel growth [34], and the mature form of Vegfd is also effective in inducing angiogenesis [35]. In our study, we also found Vegfb, Vegfc, and Vegfd were more highly expressed in ABM macrophages than in LBM macrophages, whose trends were consistent with Vegfa, which indicated that the difference in angiogenetic effects between ABM macrophages and LBM macrophages was not only due to the difference in Vegfa content but also because of the difference in the content of the Vegf family between the two. Family members of Vegf transduce signals between cells by binding to membrane-bound tyrosine-activated enzyme receptors. Vegfa often binds to Vegfr1 and Vegfr2 and Vegfb tend to bind to Vegfr1, while Vegfc and Vegfd often bind to Vegfr3 but can bind to Vegfr2 after hydrolytic cleavage of the protein to regulate cell proliferation, migration, survival, and vascular permeability to promote angiogenesis [36]. Whether the mechanism of the Vegf family binding to corresponding receptors will also cause the difference in angiogenesis between the two macrophages still needs to be explored.

In our study, we conducted the ligand–receptor analysis and predicted several ligand–receptor pairs. The interactions between multiple angiogenic factors and their corresponding receptors play great roles in the microenvironment of angiogenesis. For example, as for Vegfa, Kdr (also known as Vegfr2) appears to mediate nearly all of the observed EC responses to Vegf, while Flt1 (also known as Vegfr1) acts primarily as a ligand-binding molecule isolating Vegf from Vegfr2 signaling [37]. Nrp1, acting as a co-receptor for Vegf, amplifies Vegfa signaling by enhancing Vegfr receptor signaling in the same cell or by presenting Vegfa to the neighboring cells [38]. As for Fgf2, another strong angiogenetic factor, often binds to Fgfr1, Fgfr2, Fgfr3, and Fgfr4 to activate signaling cascades and stimulate EC proliferation, migration, and the expression of various angiogenetic growth factors [39, 40]. The interactions between ligands and receptors may affect the difference in angiogenesis between ABM and LBM macrophages, which requires us to continue exploring the mechanism in the follow-up study.

We also focused on the transcriptional analysis of the difference in macrophages at these two sites. Through SCENIC analysis, we identified six key TFs regulating the transcriptional differences between macrophages of the two sites, and Atf4 was further confirmed to be significantly highly expressed in ABM macrophages by PCR, whose predicted target genes including Vegfa. Combing the results of ATAC-seq and RNA-seq, Atf4 was also confirmed to regulate the M1 polarization of macrophages. So, we suspected Atf4 was the key TF to regulate the different expression of Vegfa between ABM macrophages and LBM macrophages by activating M1 polarization in macrophages, which was confirmed by a series of subsequent experiments. Atf4 is one of the members of the ATF/CREB family and has been revealed to be a critical regulator for bone remodeling by upregulating Vegf expression [41]. Deficiency of Atf4 exacerbates the defective angiogenesis in mice, whereas overexpression of Atf4 could ameliorate the sluggish angiogenic response [42]. The activation of Atf4-dependent osteoclast differentiation increases Vegf release from the bone matrix to promote angiogenesis through activating parathyroid hormone-related protein (PTHrP) and receptor activator of NF-κB ligand (RANKL) [41]. Some studies have shown that Atf4 may regulate macrophage polarization toward M1 phenotype by bioinformatic analysis [43, 44], but these studies have not been further confirmed by experiments. Our study applied PCR, WB and angiogenesis experiment to confirm that Atf4 could regulate M1 polarization and thus promote Vegfa expression.

There are several shortcomings in our study. First of all, although we have tried to remove the batch effects between ABM and LBM scRNA-seq, the difference in macrophage number in these two data sets still exists. Secondly, the potential signaling pathways of Atf4 leading to M1 polarization of macrophage failed to be revealed in our present study, which still needed to be explored in future. Thirdly, the present ligand–receptor analysis not only showed several potential ligands secreted by macrophages, but also did it imply potential receptors of MSCs and ECs, such as Kdr, Flt, Nrp1, and Nrp2, on which Vegfa acts. These needed further experiments to confirm which one Vegfa acts on and the mechanism behind it.

In conclusion, using scRNA-seq, we revealed ABM macrophages had stronger angiogenic effects than LBM macrophages by upregulating Vegfa expression. By SCENIC, RNA-seq, and ATAC-seq analysis, we further verified Atf4 was the critical TF to regulate the different Vegfa content by regulating M1 polarization of macrophages. Our study might provide a new idea to improve the success rate of autologous bone grafting and treatment of oral diseases.

## Supplementary Information

**Additional file 1: Figure S1. **Quality control of ABM scRNA-seq data. (A) Distribution of cells from four samples of ABM in the tSNE dimension showing no significant batch effect. (B) The violin plots showing the distribution of feature_RNA, count_RNA, ercc percentage and mitochondrial RNA percentage after quality control. (C) The variable feature plot showing the expression levels of 18445 genes in all cells. The 2000 genes with the most variable value were labeled red, and the top 10 genes were marked. (D) Heatmap of 11 cell subtypes. Top 5 genes with the highest expression in each subtype were identified and compared between the subtypes. **Figure S2. **Process of LBM scRNA-seq data and polarization status difference between the two. (A) Cells identified by scRNA-seq were visualized with UMAP. (B) Macrophage cluster was identified according to macrophage marker genes and was visualized with UMAP. (C) ABM and LBM macrophage clusters were combined and batch effects were removed. (D) Recombined macrophage groups were visualized with Tsne. (E) Expression of Arg1, Cd86, Tnf, Nos2 in ABM macrophages and LBM macrophages. ABM, alveolar bone marrow; LBM, long bone marrow. **Figure S3. **The landscape of genomic chromatin accessibility. (A) The correlative heatmaps of untreated BMDM and LPS-stimulated (M1) BMDM in RNA-seq (upper) and ATAC-seq (lower). (B) The distribution of function regions of different peaks. (C) The location distribution of different peaks distance TSS. (D) Accessible chromatin peak annotation.

## Data Availability

All data generated or analyzed during this study were included either in this article methods section. Other data that support the findings of this study are available from the corresponding author upon reasonable request.
