# Supplementary material for: Atf4 regulates angiogenic differences between alveolar bone and long bone macrophages by regulating M1 polarization, based on single-cell RNA sequencing, RNA-seq and ATAC-seq analysis
Source: J Transl Med. 2023 Mar 14;21:193. doi: 10.1186/s12967-023-04046-1 (PMC10012539; doi:10.1186/s12967-023-04046-1)
Supplement: Supplementary file 1 — Additional file 1: Figure S1. Quality control of ABM scRNA-seq data. (A) Distribution of cells from four samples of ABM in the tSNE dimension showing no significant batch effect. (B) The violin plots showing the distribution of feature_RNA, count_RNA, ercc percentage and mitochondrial RNA percentage after quality control. (C) The variable feature plot showing the expression levels of 18445 genes in all cells. The 2000 genes with the most variable value were labeled red, and the top 10 genes were marked. (D) Heatmap of 11 cell subtypes. Top 5 genes with the highest expression in each subtype were identified and compared between the subtypes. Figure S2. Process of LBM scRNA-seq data and polarization status difference between the two. (A) Cells identified by scRNA-seq were visualized with UMAP. (B) Macrophage cluster was identified according to macrophage marker genes and was visualized with UMAP. (C) ABM and LBM macrophage clusters were combined and batch effects were removed. (D) Recombined macrophage groups were visualized with Tsne. (E) Expression of Arg1, Cd86, Tnf, Nos2 in ABM macrophages and LBM macrophages. ABM, alveolar bone marrow; LBM, long bone marrow. Figure S3. The landscape of genomic chromatin accessibility. (A) The correlative heatmaps of untreated BMDM and LPS-stimulated (M1) BMDM in RNA-seq (upper) and ATAC-seq (lower). (B) The distribution of function regions of different peaks. (C) The location distribution of different peaks distance TSS. (D) Accessible chromatin peak annotation. [file 12967_2023_4046_MOESM1_ESM.docx]

**Table S1. Sequence of primers.**

| Gene | Forward primer (5’-3’) | Reverse primer (5’-3’) |
| --- | --- | --- |
| mouse Gapdh | CTACACTGAGGACCAGGTTGTCT | TTGTCATACCAGGAAATGAGCTT |
| mouse Vegfa | TGACGGACAGACAGACAGACAC | ACGGCTACTACGGAGCGAGAAG |
| mouse Tnf | CAGGCGGTGCCTATGTCTC | CGATCACCCCGAAGTTCAGTAG |
| mouse Ccl2 | TTAAAAACCTGGATCGGAACCAA | GCATTAGCTTCAGATTTACGGGT |
| mouse Tgfb1 | GCAGTGGCTGAACCAAGGA | AGCAGTGAGCGCTGAATCG |
| mouse Vegfb | GCAACACCAAGTCCGAATG | CTGGCTTCACAGCACTCTCC |
| mouse Vegfc | GAGGTCAAGGCTTTTGAAGGC | CTGTCCTGGTATTGAGGGTGG |
| mouse Vegfd | TTGAGCGATCATCCCGGTC | GCGTGAGTCCATACTGGCAAG |
| mouse Fgf2 | GCGACCCACACGTCAAACTA | TCCCTTGATAGACACAACTCCTC |
| mouse Cebpa | CAAGAACAGCAACGAGTACCG | GTCACTGGTCAACTCCAGCAC |
| mouse Klf3 | GAGTACCCCTGTCTATGCCG | CGACAAGGAAACCATGAGAGG |
| mouse Atf3 | GAGGATTTTGCTAACCTGACACC | TTGACGGTAACTGACTCCAGC |
| mouse Atf4 | AAGGAGGAAGACACTCCCTCT | CAGGTGGGTCATAAGGTTTGG |
| mouse Fosl2 | CACGCCGAGTCCTACTCCA | GTGGGCTGTACCATCCACTG |
| mouse Klf4 | GTGCCCCGACTAACCGTTG | GTCGTTGAACTCCTCGGTCT |


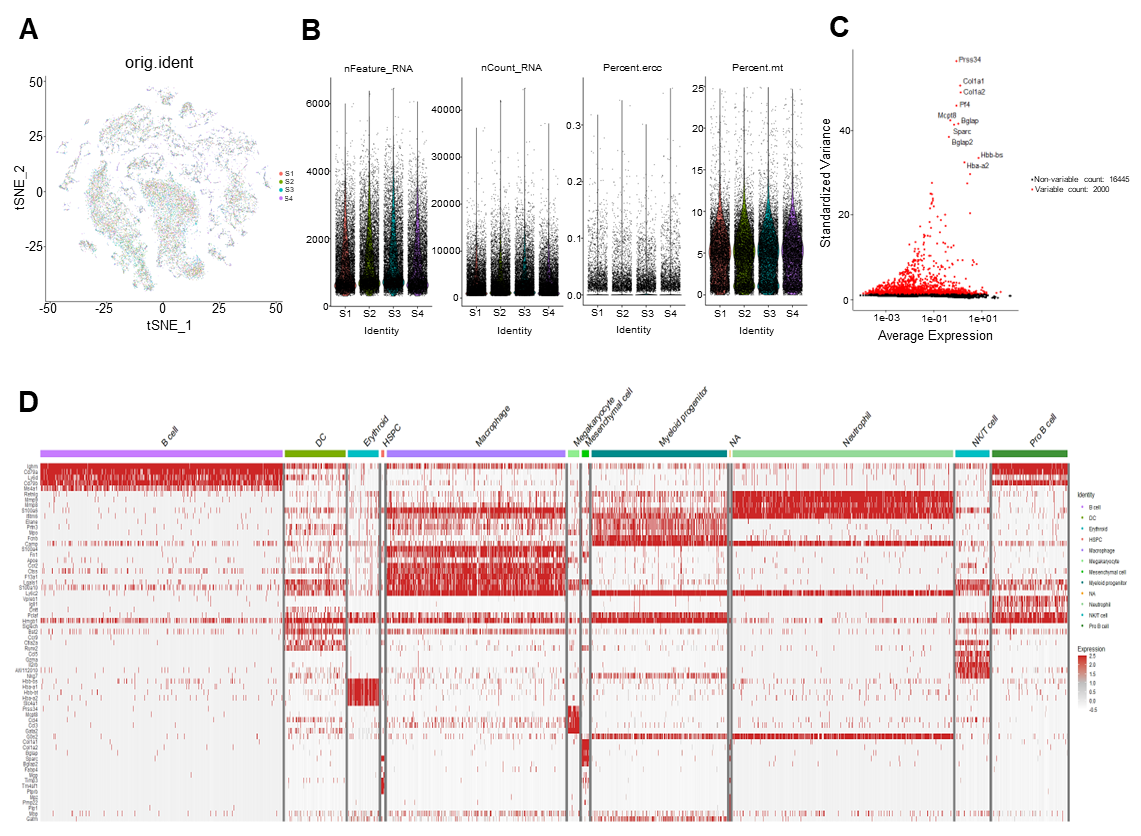


**Figure S1. Quality control of ABM scRNA-seq data.** (A) Distribution of cells from four samples of ABM in the tSNE dimension showing no significant batch effect. (B) The violin plots showing the distribution of feature_RNA, count_RNA, ercc percentage and mitochondrial RNA percentage after quality control. (C) The variable feature plot showing the expression levels of 18445 genes in all cells. The 2000 genes with the most variable value were labeled red, and the top 10 genes were marked. (D) Heatmap of 11 cell subtypes. Top 5 genes with the highest expression in each subtype were identified and compared between the subtypes.


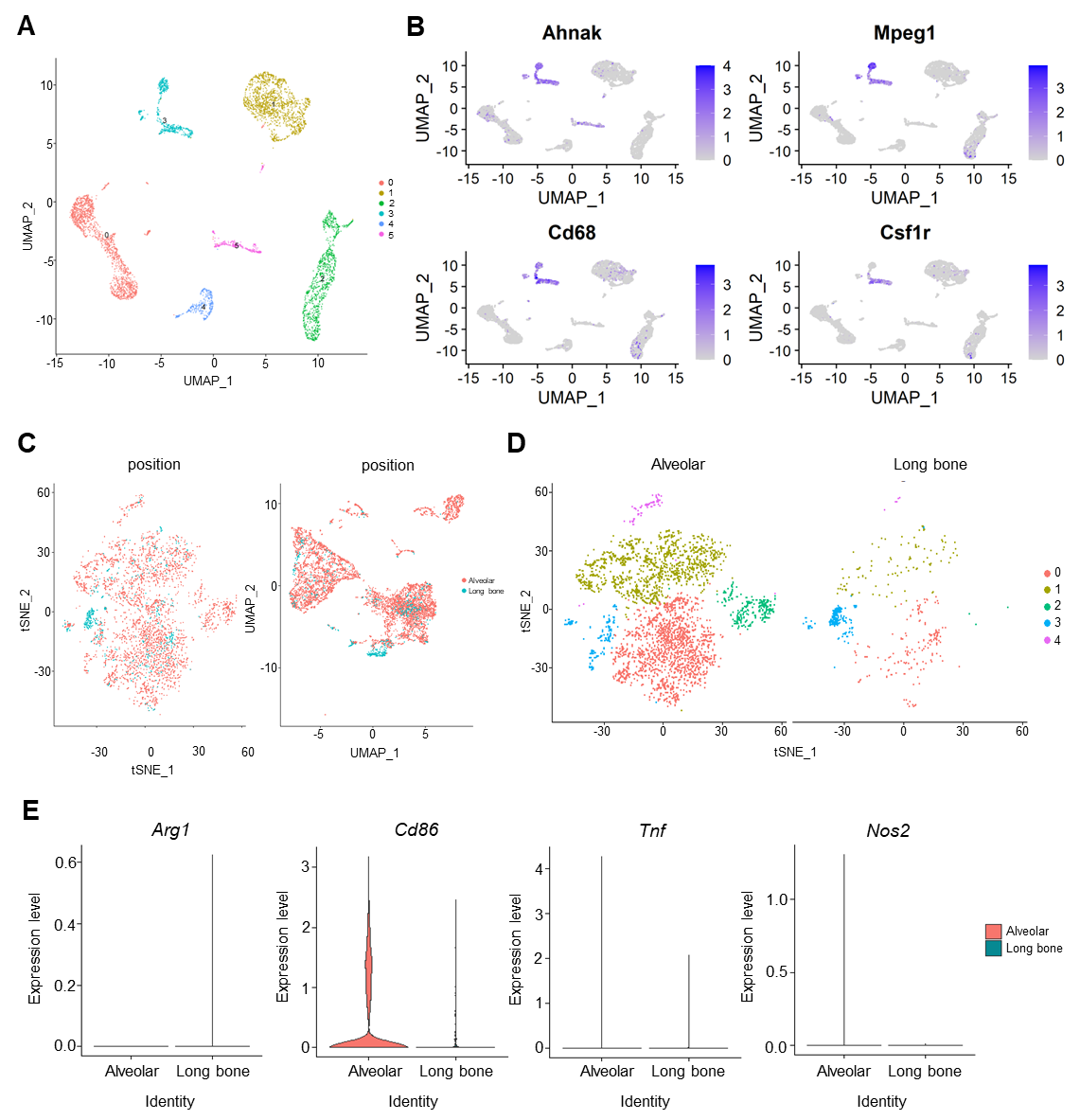


**Figure S2. Process of LBM scRNA-seq data and polarization status difference between the two.** (A) Cells identified by scRNA-seq were visualized with UMAP. (B) Macrophage cluster was identified according to macrophage marker genes and was visualized with UMAP. (C) ABM and LBM macrophage clusters were combined and batch effects were removed. (D) Recombined macrophage groups were visualized with Tsne. (E) Expression of Arg1, Cd86, Tnf, Nos2 in ABM macrophages and LBM macrophages. ABM, alveolar bone marrow; LBM, long bone marrow.


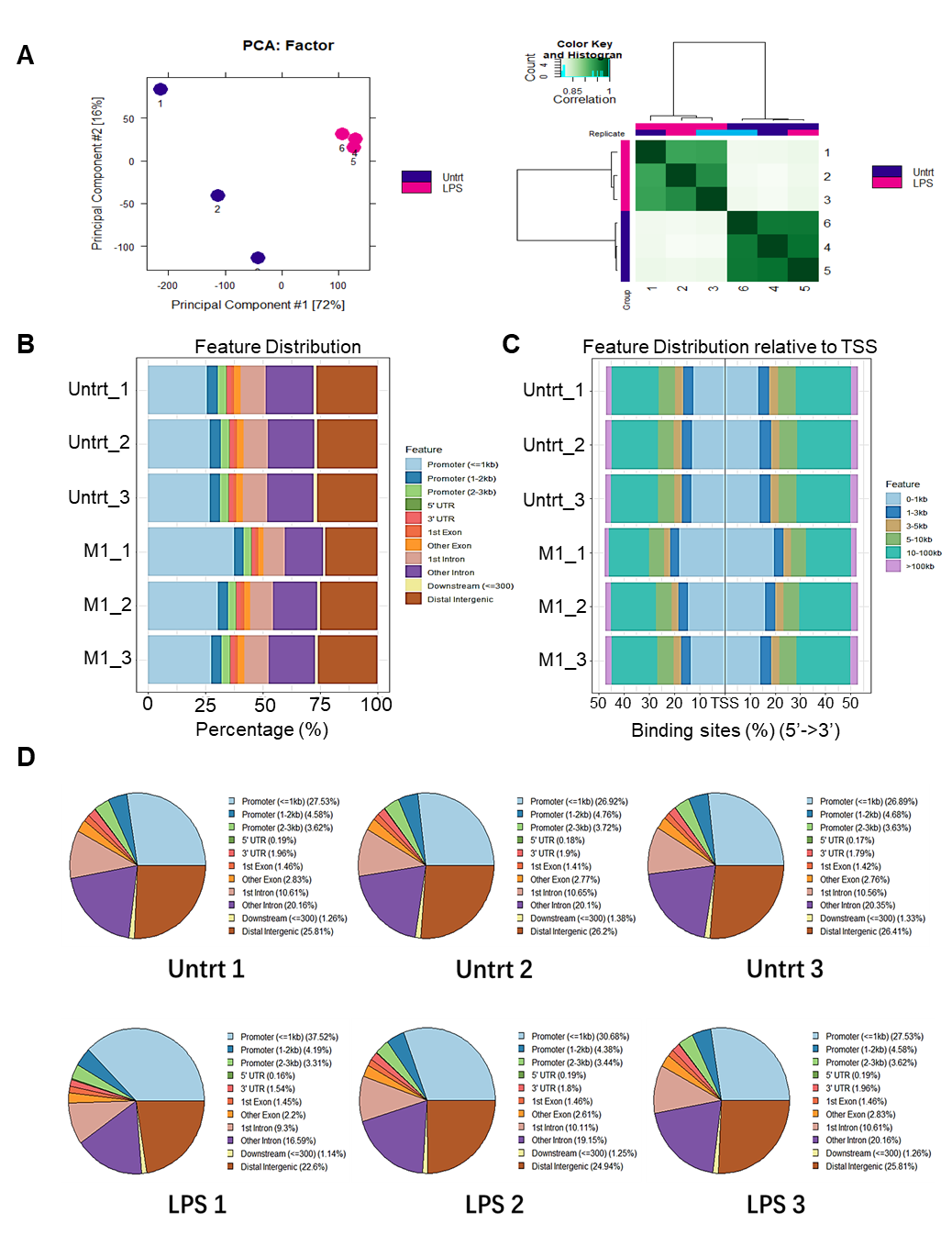


**Figure S3. The landscape of genomic chromatin accessibility.** (A) The correlative heatmaps of untreated BMDM and LPS-stimulated (M1) BMDM in RNA-seq (upper) and ATAC-seq (lower). (B) The distribution of function regions of different peaks. (C) The location distribution of different peaks distance TSS. (D) Accessible chromatin peak annotation.
